# Daily flow prediction of the Huayuankou hydrometeorological station based on the coupled CEEMDAN–SE–BiLSTM model

**DOI:** 10.1038/s41598-023-46264-z

**Published:** 2023-11-02

**Authors:** Haiyang Li, Xianqi Zhang, Shifeng Sun, Yihao Wen, Qiuwen Yin

**Affiliations:** 1 Water Conservancy College, North China University of Water Resources and Electric Power, Zhengzhou, 450046 China; 2 Collaborative Innovation Center of Water Resources Efficient Utilization and Protection Engineering, Zhengzhou, 450046 China; 3 Technology Research Center of Water Conservancy and Marine Traffic Engineering, Zhengzhou, 450046 Henan Province China

**Keywords:** Environmental sciences, Hydrology

## Abstract

Enhancing flood forecasting accuracy, promoting rational water resource utilization and management, and mitigating river disasters all hinge on the crucial role of improving the accuracy of daily flow prediction. The coupled model of Complete Ensemble Empirical Mode Decomposition with Adaptive Noise (CEEMDAN), Sample Entropy (SE), and Bidirectional Long Short-Term Memory (BiLSTM) demonstrates higher stability when faced with nonlinear and non-stationary data, stronger adaptability to various types and lengths of time series data by utilizing sample entropy, and significant advantages in processing sequential data through the BiLSTM network. In this study, in the context of predicting daily flow at the Huayuankou Hydrological Station in the lower reaches of the Yellow River, a coupled CEEMDAN–SE–BiLSTM model was developed and utilized. The results showed that the CEEMDAN–SE–BiLSTM coupled model achieved the utmost accuracy in prediction and optimal fitting performance. Compared with the CEEMDAN–SE–LSTM, CEEMDAN–BiLSTM, and BiLSTM coupled models, the root mean square error (RMSE) of this model is reduced by 42.77, 182.02, and 193.71, respectively; the mean absolute error (MAE) is reduced by 37.62, 118.60, and 126.67, respectively; and the coefficient of determination (R^2^) is increased by 0.0208, 0.1265, 0.1381.

## Introduction

In the presence of multiple climate factors and the dual influence of human activities, the prediction of river flow faces high levels of randomness, ambiguity, and uncertainty. Establishing a highly accurate flow prediction model plays a vital role in enhancing the optimization of water resource allocation within a watershed, improving the accuracy of flood forecasting, and mitigating disaster risks. Currently, the development of higher-precision flow prediction models has become a major research topic.

In recent years, several machine learning models, such as artificial neural networks (ANN), support vector machines (SVM), extreme learning machines (ELM)^1^, and Gaussian process (GP) regression, have been adopted, and models such as generalized regression neural networks (GRNN), random forest (RF) regression, and random tree (RT) based models^2^, have been widely used to predict flow in order to achieve better fitting performance. Başakin and Özger, the prediction of flow was accomplished by combining Fuzzy Time Series (FTS) with Continuous Wavelet Transform (CWT), and the findings indicated that the Wavelet Fuzzy Time Series (WFTS) method demonstrated considerably improved prediction accuracy in comparison to traditional fuzzy time series methods^3^. The improved Muskingum method was used to estimate peak flow during complete channel opening, and experimental results showed that this method had good predictive performance for flood flow evolution during the flood season^4^. Jin Baoming constructed a Backpropagation Neural Network (BPNN) model for flood prediction in a river basin, which was applied to the prediction of flow in the Shili’an section of the Minjiang River^5^. Khodakhah, Aghelpour and Hamedi, conducted a comparative analysis of various data-driven models for the prediction of monthly flow, the models considered in this study include Seasonal Autoregressive Integrated Moving Average (SARIMA), as well as machine learning models such as Least Squares Support Vector Machine (LSSVM), Adaptive Neuro-Fuzzy Inference System (ANFIS), and Group Method of Data Handling (GMDH). The study found that the SARIMA stochastic model performed well in predicting river flow under drought conditions^6^. Mehedi a Long Short-Term Memory (LSTM) neural network regression model was trained using a dataset spanning more than 80 years of daily data for univariate prediction analysis and suggested its use for real-time river discharge forecasting^7^. Hussain and Khan investigated the potential of data-driven machine learning methods, such as Multilayer Perceptron (MLP), Support Vector Regression (SVR), and Random Forest (RF), to forecast the river flow of Huzrah River in Pakistan. The analysis employed an in-situ dataset spanning the period from 1962 to 2008, enriching the machine learning algorithms and models^8^. By leveraging artificial intelligence (AI) techniques, specifically the Cascaded Correlation Neural Network (CCNN) and Random Forest (RF) models, accurate daily predictions were made for reach and river flow in two Australian river systems—the Dulhunty River and Herbert River. Based on performance accuracy, after comprehensive analysis, the CCNN model emerged as the preferred data intelligence tool for accurately predicting river stage and river flow^9^. A water flow model leveraging the Long Short-Term Memory (LSTM) architecture was developed improved by integrating the latest discharge measurements through Data Integration (DI). Despite certain limitations, deep learning-based forecasting models hold great potential due to their performance, automation, efficiency, and flexibility^10^. Liu to ensure reliability in predicting catastrophic flood years and providing long-term continuous rolling forecasts, the Empirical Mode Decomposition (EMD) algorithm was combined with the Encoder-Decoder Long Short-Term Memory (En-De-LSTM) architecture^11^. Through the comparison of Long Short-Term Memory (LSTM), Gated Recurrent Unit (GRU), and Artificial Neural Network (ANN) models, Gao Shuai suggests that GRU could be considered as the preferred approach for short-term runoff prediction^12^. The CEEMDAN–VMD–HHO–LSSVM model was constructed to predict the monthly runoff data from Manwan and Hongjiadu hydropower stations in China, which showed that the quadratic decomposition could successfully extract the complex runoff sequence information and thus significantly improve the prediction accuracy of the hybrid model (Xu et al.^13^). However, empirical modal decomposition (EMD) and variational modal decomposition (VMD) as sequence decomposition techniques cannot produce convincing forecasting models because additional information about the future flow to be predicted is introduced into the explanatory variables of the samples (Fang et al.^14^); an adaptive EEMD-ANN (AEEMD-ANN) model is proposed, which, unlike hindcasting tests, it does not use any future information; unlike traditional forecasting tests, its decomposition and forecasting model adaptively adjusts whenever new runoff information is added. It has a high forecast accuracy during flood season (Tan et al.^15^); Developed Wavelet Data-Driven Forecasting Framework (WDDFF) is a useful tool for forecasting real-world hydrologic and water resource processes, which overcomes the limitations of many earlier wavelet-based forecasting methods (Quilty and Adamowski^16^); Proposed a two-stage Disaggregated Prediction (TSDP) framework, which improves the prediction performance of watersheds lacking meteorological observations, and is more advantageous than the baseline model (Zuo et al.^17^).In summary, traditional methods for river flow prediction mainly include statistical methods and hydrological models. These methods have achieved some success to a certain extent, but due to the limitations of model assumptions, data availability, and computational power, there is still significant uncertainty in complex river flow prediction tasks. In recent years, with the significant improvement in data collection techniques and computing power, data-driven prediction methods have made significant progress in various fields. However, due to the significant spatiotemporal variability of daily flow, significant opportunities for further advancements remain in the field of daily flow prediction research.

In this study, the robustness of Complete Ensemble Empirical Mode Decomposition with Adaptive Noise (CEEMDAN) in handling nonlinear data was utilized, the strong adaptability of Sample Entropy (SE), moreover, the effectiveness of Bidirectional Long Short-Term Memory (BiLSTM) neural networks in terms of efficiency was also considered, we will construct a CEEMDAN–SE–BiLSTM coupled model using the “decomposition-reconstruction-ensemble” approach. Firstly, the data will be decomposed using the CEEMDAN method, which fully integrates empirical mode decomposition and adaptive noise. Then, the reconstructed river flow data will be quantified in terms of time series complexity using Sample Entropy (SE). Finally, the actual river flow data measured at the Huayuankou Water Station will be used to train and validate the BiLSTM model. The model employs a multilevel feature fusion that integrates CEEMDAN, SE features and BiLSTM networks. This multilevel fusion makes full use of the information at different levels, thus improving the performance of the model. By comparing with other deep learning models, the proposed coupled model in this study demonstrates higher accuracy and better stability.

## Research methodology

### Complete ensemble empirical mode decomposition with adaptive noise

**Figure 1:**
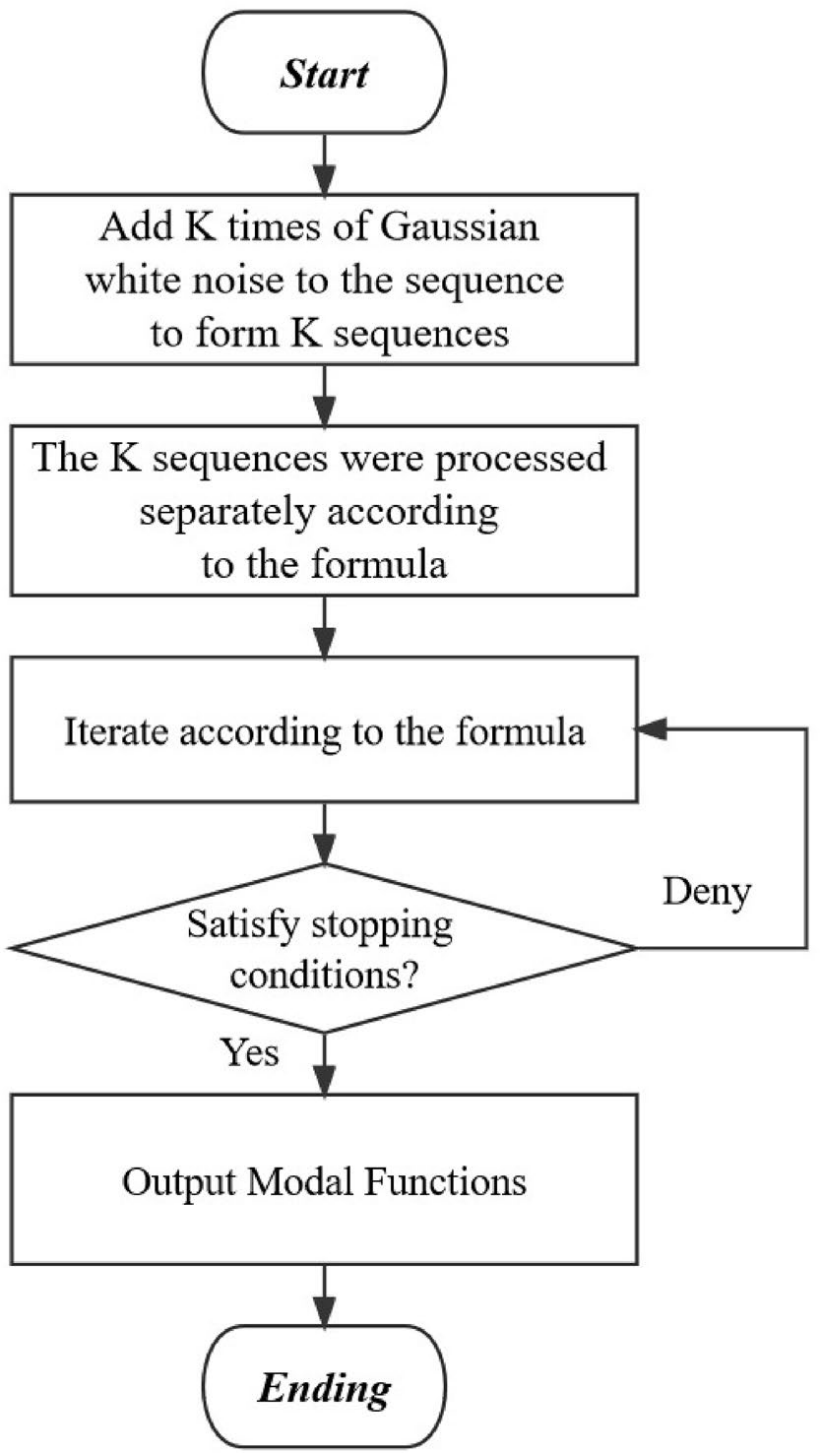
the flow chart of CEEMDAN.

CEEMDAN is a further improvement on EMD and EEMD^18^. Unlike CEEMD, which adds positive and negative white noise, CEEMDAN introduces adaptive white noise^19^. In each stage, the IMF is calculated and then averaged to obtain the final IMF sequences. Compared to the EMD and EEMD algorithms, CEEMDAN not only effectively addresses the issue of mode mixing in daily river flow, but also significantly reduces the problem of residual white noise in daily flow^20^. Additionally, this approach mitigates the challenge of alignment discrepancies in the final ensemble average that may arise due to variations in the decomposition results of each group of Intrinsic Mode Functions (IMF) within CEEMD^21^. The decomposition process of daily flow is shown in Fig. 1.

### Sample entropy

SE is an improved method based on approximate entropy utilized for assessing the complexity of non-stationary time series^22^. It indicates the likelihood of new information emerging in the daily flow time series. The more complex the daily flow time series, the larger the corresponding SE. Compared to approximate entropy, SE has advantages such as data length independence, better consistency, and simplicity of computation^23^. By using the SE algorithm to calculate the entropy values of each IMF component obtained from the decomposition of the daily flow series, it becomes feasible to quantitatively evaluate the randomness of each component. Drawing upon this information, the components of the daily flow can be merged and reconstructed, resulting in high-frequency components, low-frequency components, and trend components, as a result, this reduces the number of components and enhances computational efficiency.

The calculation steps for SE for the IMF component time series {IMF(t)} = {IMF(1), IMF(2), …, IMF(n)} of daily flow with a time length of n are as follows:

1. Arrange the sequence according to the sequence number into a vector sequence with a dimension of m, X_m_(1), …, X_m_(n − m + 1), Among them:
  1 $$X_{m} \left( i \right) = \left\{ {IMF\left( i \right),IMF(i + 1), \cdots ,IMF(i + m - 1)} \right\},1 \le i \le n - m + 1$$
  these vector sequences represent the values of m consecutive IMF components starting from the i-th point.
2. The distance between vectors X_m_(i) and X_m_(j) is determined by calculating the absolute value of the maximum difference between the corresponding elements of the two vectors. That is:
  2 $${\text{d}}\left[ {X_{m} (i),X_{m} (j)} \right] = \mathop {\max }\limits_{0 \le k \le m - 1} \left| {IMF(i + k) - IMF(j + k)} \right|$$
3. For a given X_m_(i), count the number of X_m_(j) (1 ≤ j ≤ n−m, j ≠ i) where the distance between X_m_(i) and X_m_(j) is less than or equal to r, and denote it as B_i_. For 1 ≤ i ≤ n−m, define:
  3 $$B_{i}^{m} (r) = \frac{1}{n - m - 1}B_{i}$$
  Based on this, define:
  4 $$B^{m} (r) = \frac{1}{n - m}\sum\limits_{i = 1}^{n - m} {B_{i}^{m} (r)}$$
4. Increase the dimension to m + 1, count the number of X_m+1_(i) and X_m+1_(j) (1 ≤ j ≤ n −m, j ≠ i) with a distance less than or equal to r, and denote it as A_i_. Define $A_{{\text{i}}}^{m} (r)$ as follows:
  5 $$A_{{\text{i}}}^{m} (r) = \frac{1}{n - m - 1}A_{i}$$
  Based on this, define:
  6 $$A^{m} (r) = \frac{1}{n - m}\sum\limits_{i = 1}^{n - m} {A_{i}^{m} (r)}$$
  Thus, B^m^(r) represents the probability of matching m points between two sequences under a similarity tolerance of r, while A^m^(r) represents the probability of matching m + 1 points between the two sequences under a similarity tolerance of r.
5. SE (Sample Entropy) is defined as follows:
  7 $$SE(m,r) = \mathop {\lim }\limits_{n \to \infty } \left\{ { - \ln \left[ {\frac{{A^{m} (r)}}{{B^{m} (r)}}} \right]} \right\}$$

When n is finite, the estimated sample entropy of the IMF component time series is given by:

8 $$SE(m,r,n) = - \ln \left[ {\frac{{A^{m} (r)}}{{B^{m} (r)}}} \right]$$

Calculate the SE for all IMF components of the daily flow using the aforementioned steps, and then merge and reconstruct the IMF components based on their respective SE values.

### Bidirectional long short-term memory

The Bidirectional Long Short-Term Memory network (BiLSTM) is an enhanced version derived from the Long Short-Term Memory (LSTM) network^24^, LSTM network, in itself, belongs to the category of Recurrent Neural Networks (RNN)^25^. Compared to traditional Backpropagation (BP) neural networks, RNNs can utilize temporal information. However, recurrent Neural Networks (RNNs) frequently encounter challenges such as the vanishing or exploding gradient problem when dealing with long-range dependencies between distant nodes. LSTM networks, on the other hand, can better preserve information from distant nodes and exhibit improved performance on longer temporal data^26^. Every LSTM unit comprises three gate structures: the forget gate, input gate, and output gate^27^. The formulas for the gate structures, hidden layer outputs, and cell state transition process in an LSTM unit are as follows:

9 $$f_{t} = \sigma \left( {W_{f} \cdot \left[ {h_{t - 1} ,x_{t} } \right] + b_{f} } \right)$$

10 $$i_{t} = \sigma \left( {W_{i} \cdot \left[ {h_{t - 1} ,x_{t} } \right] + b_{i} } \right)$$

11 $$\widetilde{{C_{t} }} = \tanh \left( {W_{c} \cdot \left[ {h_{t - 1} ,x_{t} } \right] + b_{c} } \right)$$

12 $$C_{t} = f_{t} * C_{t - 1} + i_{t} * \widetilde{{C_{t} }}$$

13 $$o_{t} = \sigma \left( {W_{o} \left[ {h_{t - 1} ,x_{t} } \right] + b_{o} } \right)$$

14 $$h_{t} = o_{t} * \tanh (C_{t} )$$

In the equations, $x_{t}$ represents the input time series data of daily streamflow. $f_{t}$, $i_{t}$, and $o_{t}$ represent the outputs of the forget gate, input gate, and output gate, respectively. $W_{f}$, $W_{i}$, and $W_{o}$ are the weight matrices corresponding to the three gates, while $b_{f}$, $b_{i}$, and $b_{o}$ are the respective bias units. $\sigma$ represents the sigmoid function, and tanh represents the hyperbolic tangent function. The symbol “*” denotes the inner product operation. $\widetilde{{C_{t} }}$ represents the candidate vector created through the tanh layer, while $W_{c}$ and $b_{c}$ correspond to the weight matrix and bias unit of that layer. $C_{t}$ represents the cell state, and $h_{t}$ represents the hidden state.

**Figure 2:**
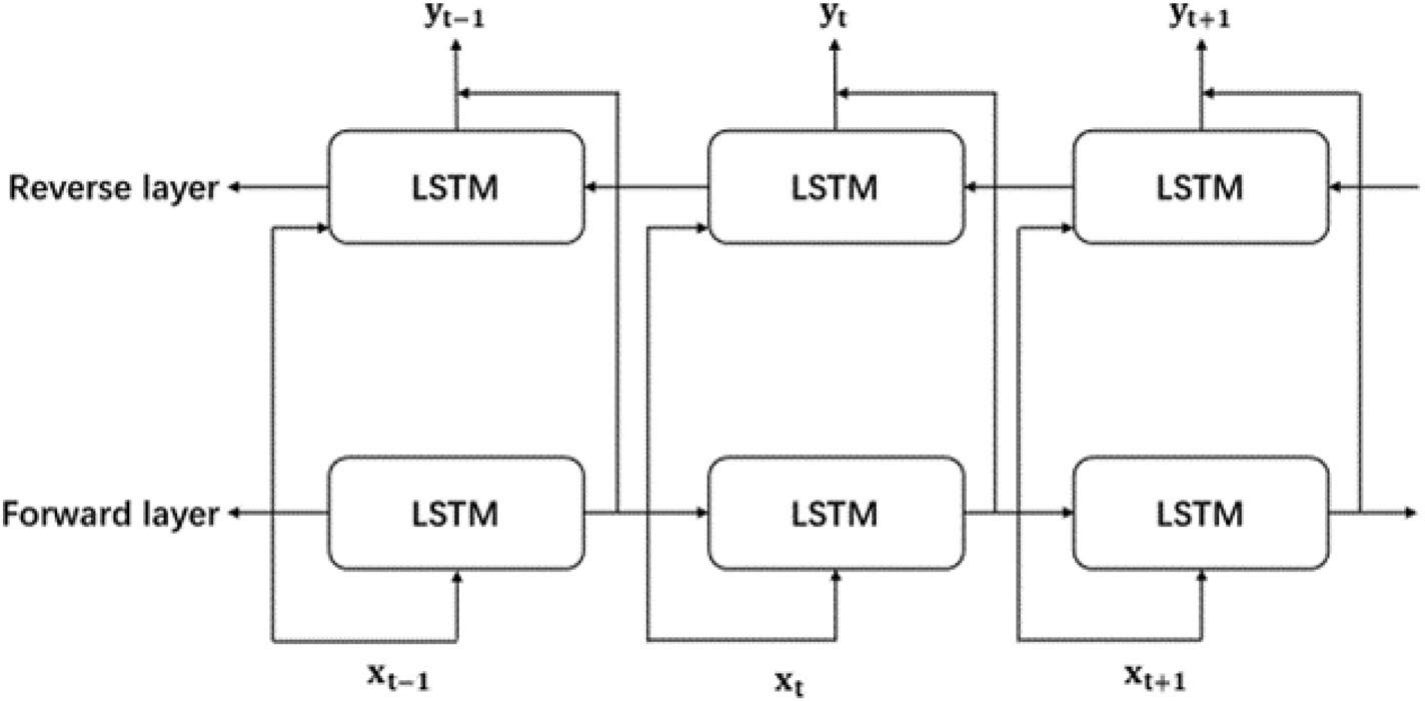
BiLSTM structure diagram.

However, LSTM only takes into account the information from the forward sequence when predicting the results in a neural network, making it difficult to capture the content of backward data^28^. The emergence of Bidirectional Long Short-Term Memory (BiLSTM) addresses this issue of lacking attention to backward information. The term "bidirectional" means that BiLSTM consists of both an LSTM unit is divided into a forward LSTM unit and a backward LSTM unit^29^, with each LSTM unit being consistent with the LSTM structure mentioned earlier. The forward and backward units operate independently of each other^30^. Figure 2 illustrates the architecture of the BiLSTM network. Existing research indicates that BiLSTM outperforms LSTM in predicting results on time series data.

### The CEEMDAN–SE–BiLSTM coupled model

#### Model construction

**Figure 3:**
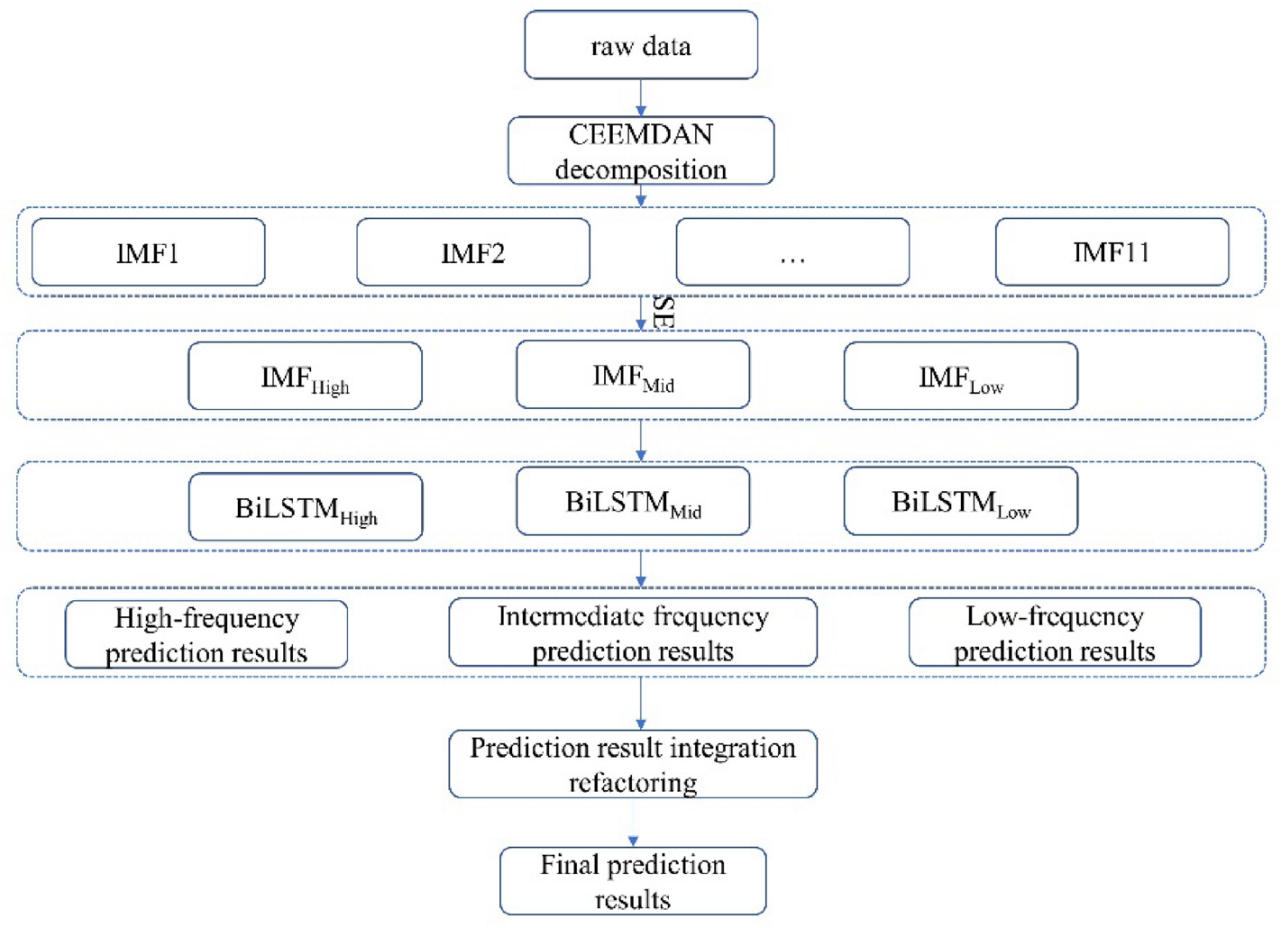
CEEMDAN–SE–BiLSTM flowchart.

To address the non-stationarity of daily streamflow time series, a coupled CEEMDAN–SE–BiLSTM model was established, and its workflow is illustrated in Fig. 3. The specific modeling steps are outlined below:

1. *CEEMDAN Decomposition* The original daily streamflow data is decomposed using CEEMDAN, resulting in IMF components of the time series.
2. The IMF components obtained from the decomposition of CEEMDAN are integrated and reconstructed using the SE algorithm, resulting in high-frequency, mid-frequency, and low-frequency IMF components.
3. *Data Division for Training and Prediction* The IMF components corresponding to the first 90% of the daily streamflow data are utilized as training data for the BiLSTM neural network, while the IMF components associated with the last 10% of the daily streamflow data are employed as prediction data for the BiLSTM neural network.
4. *Data Normalization* To mitigate the influence of significant variations in input data on prediction accuracy, both the training data and prediction data are normalized within the range of [0, 1]. The normalization formula employed is as follows:
  15 $$y = \frac{{x - x_{\min } }}{{x_{\max } - x_{\min } }}$$
  In the formula, x represents the original value at time t; x_min_ represents the minimum value of the sequence; x_max_ represents the maximum value of the sequence; y represents the normalized value at time t.
5. *Training the BiLSTM Neural Network* By fine-tuning the network parameters of the BiLSTM neural network, the training performance on the training data is enhanced, thereby improving the prediction accuracy of the BiLSTM neural network for the IMF components of the daily flow.
6. *BiLSTM Neural Network Prediction* The optimized BiLSTM neural network is utilized for predicting the IMF components corresponding to the first 90% of the daily flow.
7. *Prediction Data Reconstruction* The predicted IMF components are subjected to inverse normalization, and the reconstructed values of the last 10% of the daily flow are obtained.

#### Model accuracy evaluation criteria

To better reflect the predictive performance of the CEEMDAN–SEBiLSTM coupled model on daily streamflow, three classic statistical metrics were selected for evaluation in this study. The quantitative evaluation criteria employed in this study are Mean Absolute Error (MAE), Root Mean Square Error (RMSE), and Coefficient of Determination (R^2^). The calculation formulas for these metrics are as follows:

16 $$RMSE = \sqrt {\frac{1}{n}\sum\limits_{i = 1}^{n} {(Q_{i} - Q_{i}^{*} )^{2} } }$$

17 $$MAE = \frac{1}{n}\sum\limits_{i = 1}^{n} {\left| {Q_{i} - Q_{i}^{*} } \right|}$$

18 $$R^{2} = 1 - \frac{{\sum\limits_{i = 1}^{n} {(Q_{i} - Q_{i}^{*} )^{2} } }}{{\sum\limits_{i = 1}^{n} {(Q_{i} - \overline{Q} )^{2} } }}$$

Among them, $Q_{i}$ represents the measured daily flow data, $Q_{i}^{*}$ represents the predicted daily flow data, and n represents the number of time series.

## Case study analysis

### Data source

**Figure 4:**
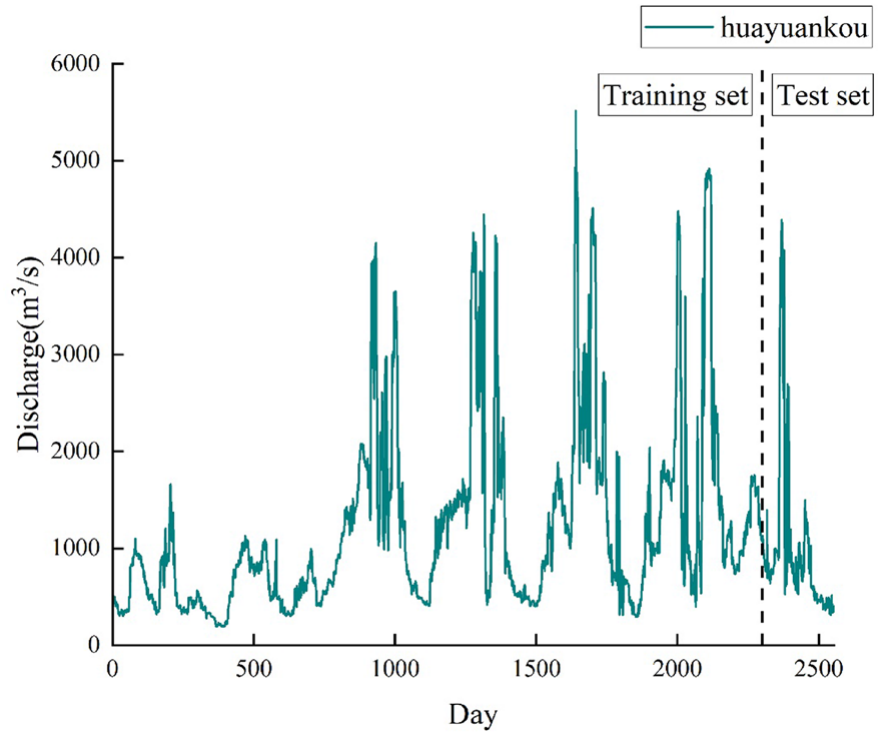
Daily flow sequence of Huayuankou from 2016 to 2022.

The Huayuankou Hydrological Station assumes significant responsibilities, including water resource management in the lower reaches of the Yellow River, regional water resource development, and analysis of hydrological and water resource dynamics. The hydrological data at the station are well-preserved. For this study, daily measured flow data from the Huayuankou Hydrological Station for the years 2016–2022 were used as the research object. The variation curve is shown in Fig. 4.

### Model validation and comparative analysis

It can be observed that the daily flow at Huayuankou Hydrological Station exhibits highly nonlinear and nonstationary characteristics. The extreme values of daily flow primarily manifest during the flood season, exhibiting notable temporal variation and intricate complexity. Following the steps of CEEMDAN decomposition mentioned earlier, the daily flow data from 2016 to 2022 at Huayuankou station was subjected to CEEMDAN decomposition.

**Figure 5:**
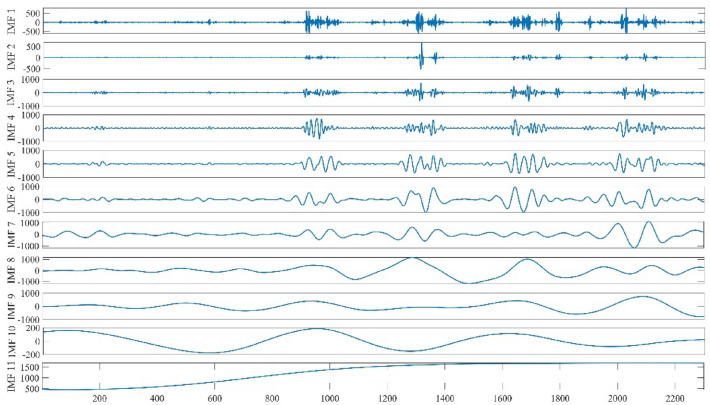
Huayuankou daily traffic data CEENDAN decomposition from 2016 to 2022.

By examining Fig. 5, it is evident that the flow sequence is decomposed into 10 Intrinsic Mode Function (IMF) components along with a corresponding residue. Among these components, the initial IMF components demonstrate the highest volatility and frequency, and shortest wavelength, while the amplitudes, frequencies, and wavelengths gradually decrease in the subsequent IMF components.

**Figure 6:**
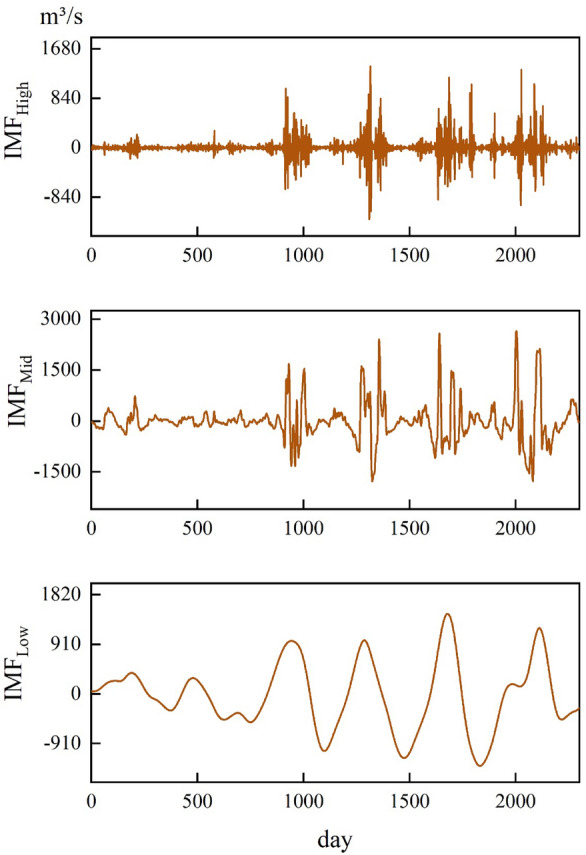
High, mid and low frequency IMF component diagram.

Subsequently, the obtained IMF components are integrated and reconstructed using the SE algorithm, resulting in three new IMF components: high-frequency, mid-frequency, and low-frequency. The new IMF component plots are shown in Fig. 6.

As depicted in the figure, the IMF components after integration and reconstruction using the SE algorithm exhibit reduced fluctuations. Moreover, this approach not only substantially decreases the computational complexity of the prediction but also enhances the accuracy and stability of the model.

### Daily flow prediction

When predicting the daily flow of Huayuankou using the BiLSTM network, it is essential to partition the data into training and testing samples. The training sample consists of the initial 90% of the IMF data, whereas the testing sample comprises the remaining 10% of the IMF data.

At the same time, the parameters set for the BiLSTM network model have a significant impact on the accuracy of the combined prediction model. The purpose of adjusting these parameters is to improve the accuracy of the prediction model. In this study, the BiLSTM network employed the tanh activation function, the Adam optimizer, and the RMSE loss function. The Dropout method was used to prevent overfitting. The model’s hyperparameters that require adjustment encompass the number of input, output, and hidden layer nodes, training iterations, and Dropout rate. In this study, a series of trial-and-error experiments were performed to identify the optimal hyperparameters. Trial-and-error experiments involve fixing the values of other hyperparameters and conducting multiple iterations to compare the predicted values with the actual values, resulting in the determination of the hyperparameters as shown in Table 1.

**Table 1:** BiLSTM network hyperparameters.

Parameter Name	Parameter size
Number of layers in the hidden layer	2
Number of nodes in the hidden layer	64
Batch-size	128
Dropout	0.1
Training times	250

**Figure 7:**
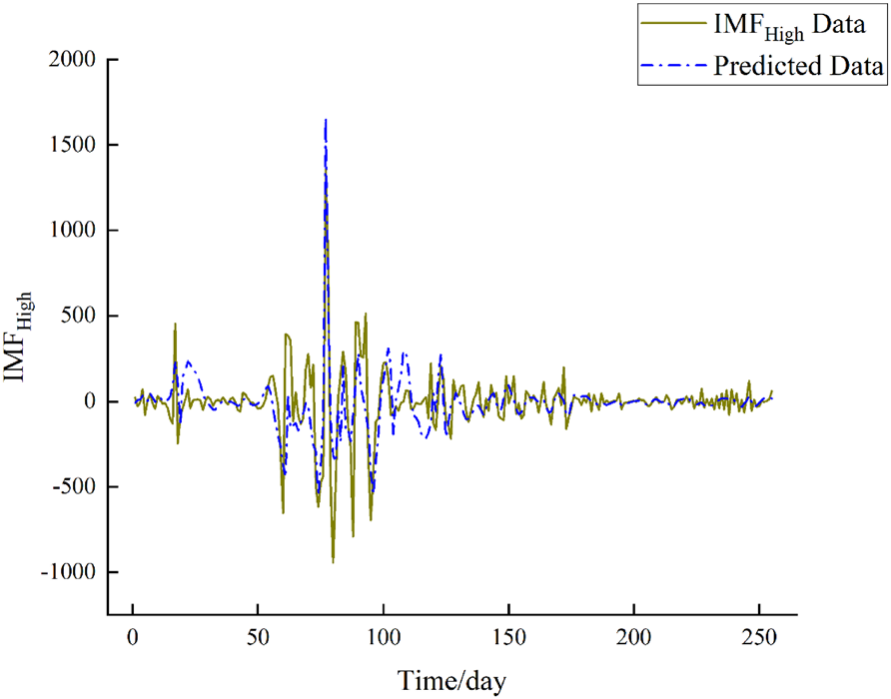
IMF_High_ forecast chart.

**Figure 8:**
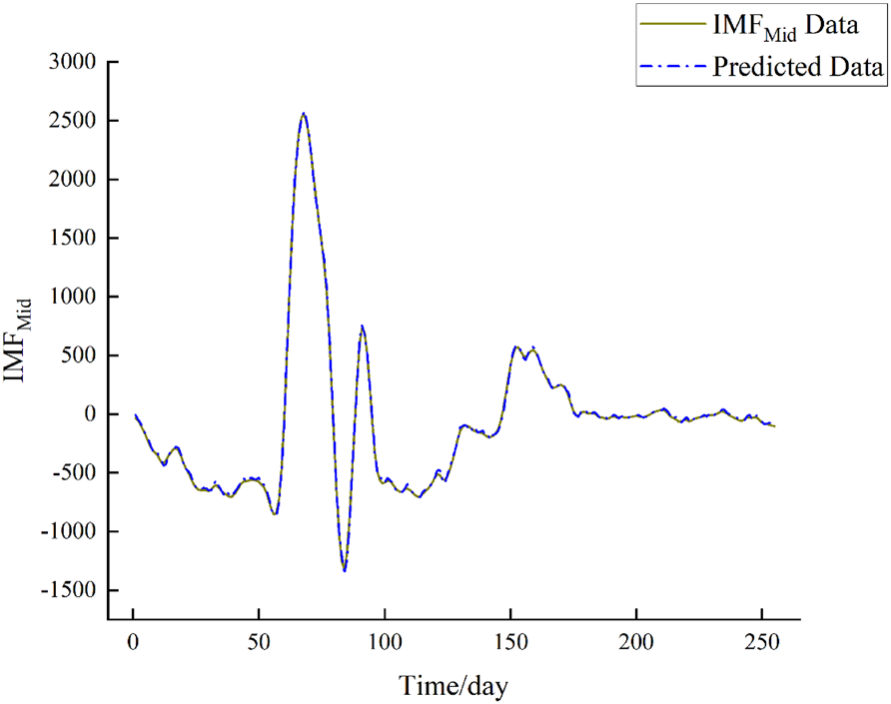
IMF_Mid_ forecast chart.

**Figure 9:**
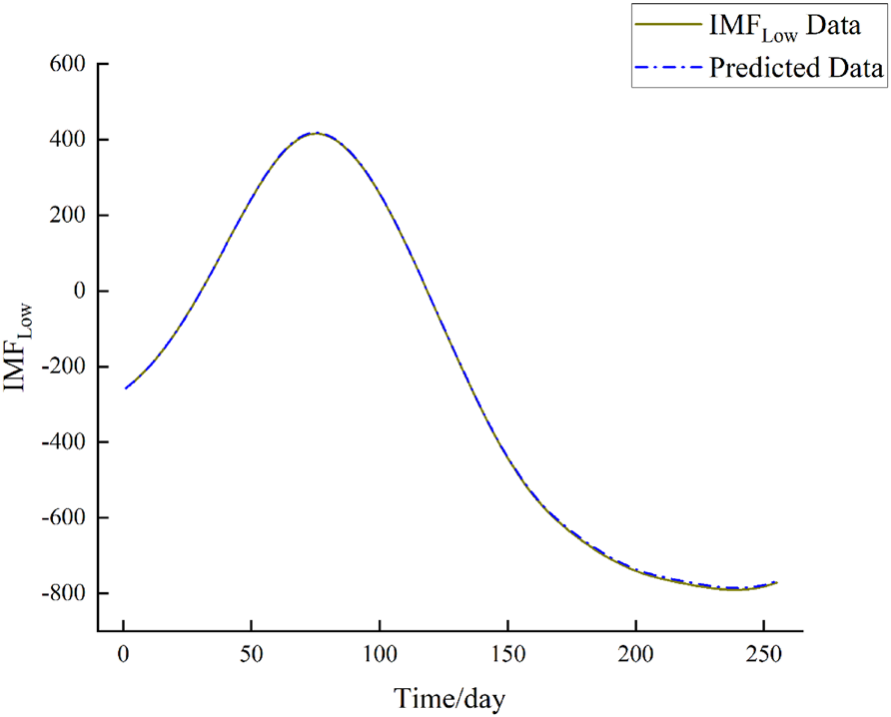
IMF_Low_ forecast chart.

Utilizing the preceding steps, the BiLSTM network is employed to forecast the three IMF components (IMF_High_, IMF_Mid_, IMF_Low_) of the Huayuankou Station. The initial 90% of the IMF data serves as training samples, while the remaining 10% is designated as testing samples. Specifically, the first 2300 data points are allocated for training, followed by the subsequent 255 data points for prediction. The prediction outcomes are illustrated in Figs. 7, 8 and 9.

From the above figures, upon observation, it can be noted that the prediction performance of the IMFHigh component exhibits a slight decline, indicating a higher level of non-stationarity in the IMF_High_ component. Conversely, the prediction performance of the IMFMid and IMFLow components demonstrates improvement, indicating a lower level of non-stationarity in these components.

**Figure 10:**
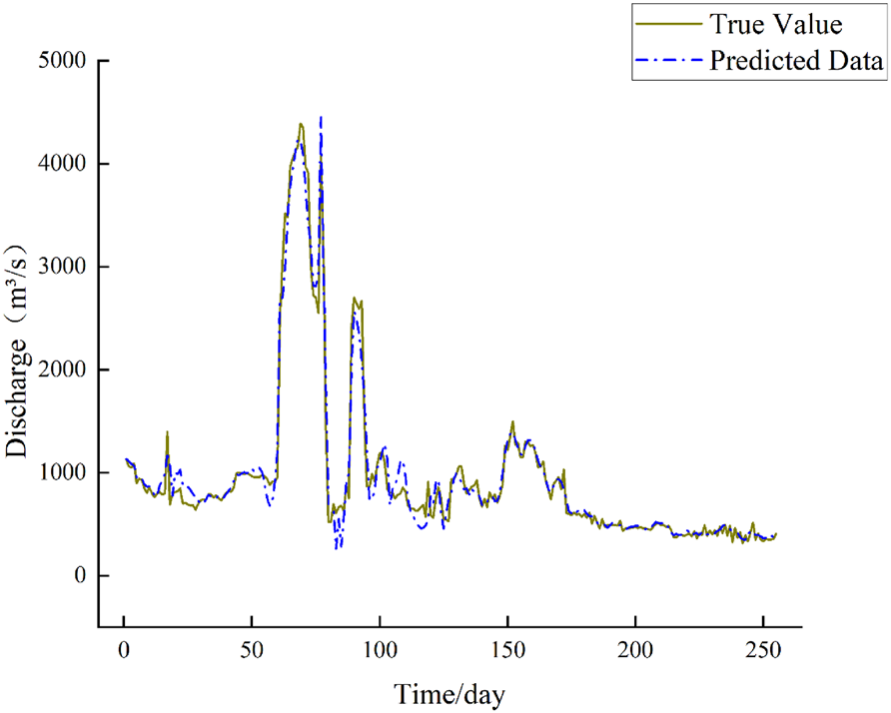
Huayuankou final traffic forecast.

By integrating and reconstructing the above prediction results, the final prediction outcome for the Huayuankou Station is obtained, as shown in Fig. 10.

From Figs. 7, 8, 9, and 10, by observing the results, it becomes evident that the daily streamflow predictions of the coupled CEEMDAN–SE–BiLSTM model exhibit a commendable alignment with the actual values, indicating a high level of model fit. According to Table 2, the IMF_High_ component exhibits larger errors, suggesting that the IMF_High_ data still possesses significant non-stationarity. On the other hand, the errors for the IMF_Mid_ and IMF_Low_ components are very small, showing a good alignment with the original data. Overall, the errors remain within a reasonable range.

**Table 2:** Error analysis of individual components.

Error type	IMF_High_	IMF_Mid_	IMF_Low_
RMSE	149.71	17.62	3.11
MAE	88.58	13.72	2.49
R^2^	0.4217	0.9982	0.9999

## Discussion

The daily streamflow data of the Huayuankou hydrological station from 2016 to 2022 was decomposed using CEEMDAN, and the decomposition results are illustrated in Fig. 5. It can be observed that IMF1 of the Huayuankou station has the highest frequency, largest amplitude, shortest wavelength, and the smallest periodicity. The stability of IMF2 to IMF7 gradually increases, while IMF8 to IMF10 exhibit relatively stable fluctuations. Next, based on the SE algorithm, the IMF components are integrated and reconstructed to obtain three new IMF components: IMF_High_, IMF_Mid_, and IMF_Low_. The new IMF component plot is shown in Fig. 6. It can be seen that after the integration and reconstruction using the SE algorithm, the three IMF components, IMF_High_, IMF_Mid_, and IMF_Low_, exhibit reduced fluctuations. This not only significantly reduces the computational burden for predictions but also improves the accuracy and stability of the model.

Using BiLSTM, the decomposed and integrated data from CEEMDAN for the three components of the Huayuankou hydrological station were simulated and predicted. The predicted results were summed to obtain the daily streamflow forecast for the Huayuankou station. The training set consisted of a total of 2300 data points from January 2016 to March 2022, while the prediction set comprised 255 data points from April to December 2022. The obtained results are depicted in Fig. 10.

**Figure 11:**
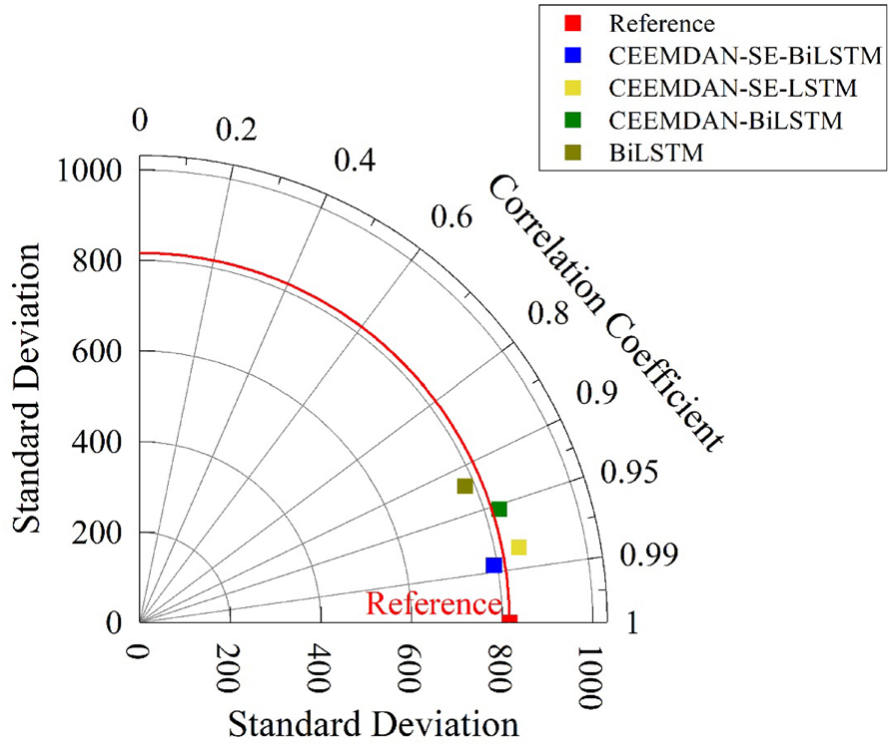
Comparison chart of accuracy of individual models.

In order to verify the finiteness, accuracy and robustness of the CEEMDAN–SE–BiLSTM coupled model for the prediction of daily runoff, the prediction results of the CEEMDAN–SE–BiLSTM coupled model were compared with those of the CEEMDAN–SE–LSTM, CEEMDAN–BiLSTM, and BiLSTM coupled models as shown in Fig. 11, and the error analyses of the individual models are shown in Table 3.

**Table 3:** Error comparison table for each model.

Models	RMSE (m^3^/s)	MAE (m^3^/s)	R^2^
CEEMDAN–SE–BiLSTM	139.73	87.67	0.9706
CEEMDAN–SE–LSTM	182.50	125.29	0.9498
CEEMDAN–BiLSTM	321.75	206.27	0.8441
BiLSTM	333.44	214.34	0.8325

Figure 11 reveals that the CEEMDAN–SE–BiLSTM coupled model showcases the closest alignment with the true values, displaying the most favorable fitting performance. The other models have lower accuracy compared to the model used in this study, with the following order of performance: CEEMDAN–SE–LSTM > CEEMDAN–BiLSTM > BiLSTM. According to Table 3, the CEEMDAN–SE–BiLSTM coupled model demonstrates smaller values for both root mean square error and mean absolute error compared to other coupled models, and the coefficient of determination is 0.9706, higher than that of other coupled models, approaching 1. This indicates that the CEEMDAN–SE–BiLSTM coupled model achieves the best fitting performance. This is attributed to the better stability of CEEMDAN in handling nonlinear data, the better adaptability of SE, and the efficiency and accuracy of BiLSTM, which significantly reduce the prediction errors and improve the data fitting capability. Therefore, the CEEMDAN–SE–BiLSTM coupled model can accurately simulate the complex and multi-frequency variations of streamflow during flood periods. The model and method can provide reference for hydrological prediction and related forecasting studies.

From the above comparative analysis of the prediction results, it can be concluded that the streamflow series is a non-stationary sequence, and using a single machine learning method cannot accurately capture the complex characteristics of streamflow. The CEEMDAN–SE–BiLSTM coupled model can effectively decompose complex time series, facilitate the extraction of underlying feature indicators, and enhance the learning and prediction of the BiLSTM model. This approach significantly improves the accuracy of streamflow prediction.

## Conclusion

To address the challenges posed by the nonlinear and non-stationary characteristics of daily streamflow time series, this study proposes a novel model, the CEEMDAN–SE–BiLSTM coupled model, based on the “decomposition-reconstruction-ensemble” concept. The effectiveness of this coupled model in daily streamflow prediction was evaluated using data from the Huayuankou Hydrological Station in the lower reaches of the Yellow River. Comparative analysis was performed against the prediction results of the CEEMDAN–SE–LSTM, CEEMDAN–BiLSTM, and BiLSTM coupled models, leading to the following conclusions:

1. The results of daily flow prediction at the Huayuankou Hydrological Station on the lower reaches of the Yellow River show that the coupled CEEMDAN–SE–BiLSTM model proposed in this paper has good accuracy and robustness. The decision coefficient of this model is 0.9706, which is the highest among the four models, and its RMSE and MAE are 139.73 m^3^/s and 87.67 m^3^/s, respectively, which are reduced compared with other models. This indicates that the CEEMDAN–SE–BiLSTM coupled model for daily flow prediction is feasible and can be effectively used for time series analysis in hydrology and related fields to guide the rational development and improved utilization of water resources.
2. The CEEMDAN–SE–BiLSTM coupled model proposed in this study, with its systematic approach involving data preprocessing, decomposition, reconstruction, ensemble, and prediction, offers significant benefits in terms of reducing prediction errors, enhancing data fitting capacity, and improving model stability. It can be regarded as a valuable method for enhancing and expanding short- to medium-term streamflow prediction capabilities.
3. Despite the promising applications of the CEEMDAN–SE–BiLSTM coupled model, which benefits from its effective decomposition algorithm, stable and efficient integration and reconstruction capability, and reliable prediction performance, it also has inherent limitations. One such limitation is the inability to incorporate the lag effect of physical mechanisms, such as precipitation, on streamflow, as the model solely relies on the streamflow time series as input. This aspect highlights the need for future research to address this limitation and explore ways to incorporate additional variables to enhance the model's predictive capabilities.

## Data Availability

Data and materials are available from the corresponding author upon request.
